# Cyclic Ion Mobility–Collision Activation Experiments Elucidate Protein Behavior in the Gas Phase

**DOI:** 10.1021/jasms.1c00018

**Published:** 2021-05-18

**Authors:** Charles Eldrid, Aisha Ben-Younis, Jakub Ujma, Hannah Britt, Tristan Cragnolini, Symeon Kalfas, Dale Cooper-Shepherd, Nick Tomczyk, Kevin Giles, Mike Morris, Rehana Akter, Daniel Raleigh, Konstantinos Thalassinos

**Affiliations:** † Institute of Structural and Molecular Biology, Division of Bioscience, University College London, London, WC1E 6BT, U.K.; ‡ Waters Corporation, Wilmslow SK9 4AX, U.K.; § Institute of Structural and Molecular Biology, Birkbeck College, University of London, London WC1E 7HX, U.K.; ∥ Department of Chemistry, Stony Brook University, 100 Nicolls Road, Stony Brook, New York 11794, United States

**Keywords:** ion-mobility mass spectrometry, protein unfolding

## Abstract

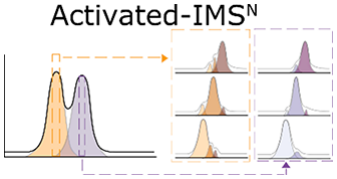

Ion mobility coupled to mass spectrometry (IM-MS) is widely used to study protein dynamics and structure in the gas phase. Increasing the energy with which the protein ions are introduced to the IM cell can induce them to unfold, providing information on the comparative energetics of unfolding between different proteoforms. Recently, a high-resolution cyclic IM-mass spectrometer (cIM-MS) was introduced, allowing multiple, consecutive tandem IM experiments (IM^n^) to be carried out. We describe a tandem IM technique for defining detailed protein unfolding pathways and the dynamics of disordered proteins. The method involves multiple rounds of IM separation and collision activation (CA): IM-CA-IM and CA-IM-CA-IM. Here, we explore its application to studies of a model protein, cytochrome C, and dimeric human islet amyloid polypeptide (hIAPP), a cytotoxic and amyloidogenic peptide involved in type II diabetes. In agreement with prior work using single stage IM-MS, several unfolding events are observed for cytochrome C. IM^n^-MS experiments also show evidence of interconversion between compact and extended structures. IM^n^-MS data for hIAPP shows interconversion prior to dissociation, suggesting that the certain conformations have low energy barriers between them and transition between compact and extended forms.

Ion mobility (IM) coupled to native mass spectrometry (MS) is a powerful tool for the interrogation of protein structure and dynamics. Native MS allows the study of intact protein ions in the gas phase by preserving native-like structures through soft ionization techniques, such as electrospray ionization (ESI).^1−4^ Native MS has proven useful for the identification of heterogeneous mixtures through the separation of *m*/*z* such as identification of protein–protein^5,6^ and protein–ligand^7,8^ interactions and large-scale changes to global protein fold.^9^

MS instruments equipped with an IM cell (IM-MS) can resolve structural isomers: more extended conformers undergo a greater number of collisions with gas molecules in the IM cell and so have a reduced mobility (*K*_0_) and higher collision cross-section (CCS) compared to ions which are more compact.^10−12^ Produced is an arrival time distribution (ATD) where the comparative intensity of different conformations can be assessed. IM-MS has allowed much greater insight into the dynamic behavior of proteins by combining the advantages of MS, such as high sensitivity for heterogeneous mixtures, with IM, which allows disambiguation of conformers through CCS, as well as reporting on their relative abundance. IM-MS has been used to probe protein conformation^13^ and protein dynamics^14^ and characterize intrinsically disordered proteins (IDPs).^15^ Protein ions can be collisionally activated (CA) to induce unfolding (CIU) and/or dissociation (CID) during injection into the IM cell.^16,17^ These techniques have been successfully applied to characterize the number of domains within a protein,^18^ antibody arm swapping,^19^ and subunit organization.^16^

IM-MS emerged as one of the best techniques to study intrinsically disordered proteins (IDPs) due to their conformational heterogeneity being a limiting factor in analyses via traditional structural biology techniques (X-ray crystallography, cryo-electron microscopy, circular dichroism, and nuclear magnetic resonance). On the other hand, IM-MS measures abundances, charge state distribution, and CCS distribution, which can be used to characterize disordered proteins.^20^ Furthermore, a variety of CA techniques can be used to probe the dynamics within the detected ensembles.

Further understanding of IDP dynamics could be elucidated using tandem IM. Tandem IM, whereby multiple rounds of IM selection take place, can be applied to the field of protein folding and dynamics. Tandem IM coupled to MS is a rapidly expanding field and has already been used in the field of proteomics.^21−23^ It was first used to increase the peak capacity and sensitivity for peptide fragmentation and was expanded to an application of studying protein unfolding and dynamics.^24,25^ The use of tandem IM was restricted to specialized research groups which had the knowledge and skills to construct such instruments *in-house*. Recently, the first commercial quadrupole time-of-flight (QToF) instrument with a cyclic IM cell (cIM) capable of tandem IM was introduced.^26^ The instrument was shown to be of value for use in native IM-MS.^26,27^ The separation performance of the system can be improved by increasing the number of passes around the cIM drift-cell. For rigid molecules, gains agree with the expected diffusion limit of resolving power;^25^ this is not necessarily the case for native protein ions which are structurally heterogeneous and may exhibit dynamic behavior on the separation time-scales.^25−27^ This system is also capable of performing multiple rounds of IM selection (IM^n^) through the use of a multidirectional array, as well as selecting subportions of ion packets for further separation and investigation.^26,27^

In this paper, we set out a methodology for a tandem IM technique for characterizing unfolding pathways of proteins, termed “slice-CA”. Slice-CA involves multiple rounds of IM separation and activation: IM-CA-IM and CA-IM-CA-IM. In the first approach (IM-CA-IM), ions enter the cIM device and complete one pass around the device. During ejection, ions can be directed “forward”, toward the detector, or “backward”, into the store region. The duration of these ejections can be timed such that ions of selected drift time range are ejected into the store. The remaining (i.e., “unselected”) ions are transferred into the ToF analyzer and detected, yielding an arrival time distribution (ATD) with a characteristic “sliced-out” feature (see Figure 2, 20–33 ms). Subsequently, selected ions are reinjected from the store into the cIM device, at increased energies, resulting in collisional activation and conformational rearrangements. Then the rearranged conformers complete an additional pass around the cIM device before being ejected to ToF and detected. This results in the ATD of rearranged conformers, which originate from the selected population (i.e., selected ATD “slice”, Figure 2, 50–100 ms). This process can be repeated multiple times, such that the populations across the original ATD are “sliced”, activated, and separated. An extension of this methodology involves activation before the first round of separation (CA-IM-CA-IM), such that transitions originating from preactivated conformers can be studied. We demonstrated this technique by further exploring the unfolding pathway of cytochrome C (CytC)^27^ and by applying the slice-CA methodology to the disordered amyloidogenic protein human islet amyloid polypeptide (hIAPP or amylin), showing interconversion of dimeric conformations under activation.

## Methods

### Sample Preparation

Equine CytC (Merck Millipore, UK) was buffer exchanged into 200 mM ammonium acetate, using Amicon Ultra centrifugal filtration units (Merck Millipore, UK), Concentration calculation was performed using the Qubit assay (Thermo Fisher), and CytC was diluted to 10 μM. IAPP was synthesized using Fmoc solid phase synthesis and purified via HPLC as previously described (supporting methods).^28^ Lyophilized IAPP samples were dissolved in 100% DMSO to a concentration of 3.2 mM, and incubated without agitation for 24 h at 37 °C and diluted immediately before data collection 100-fold in 100 mM ammonium acetate pH 7.4, to a final concentration of 32 μM. The final concentration of DMSO was 1% (v/v).

### Data Collection

Data was collected on two different cIM QToFs, a prototype instrument at Waters Corp., Wilmslow, and a commercial instrument at University College London. Samples were directly infused using nano ESI (nESI) gold-coated capillaries produced *in-house* using a Flaming-Brown P97 micropipette puller and coated using a Quorum Q150RS sputter coater. For data collection parameters for CytC, see Table S1, and for IAPP, see Table S7.

### Slice-CA (IM^n^)

**Figure 1:**
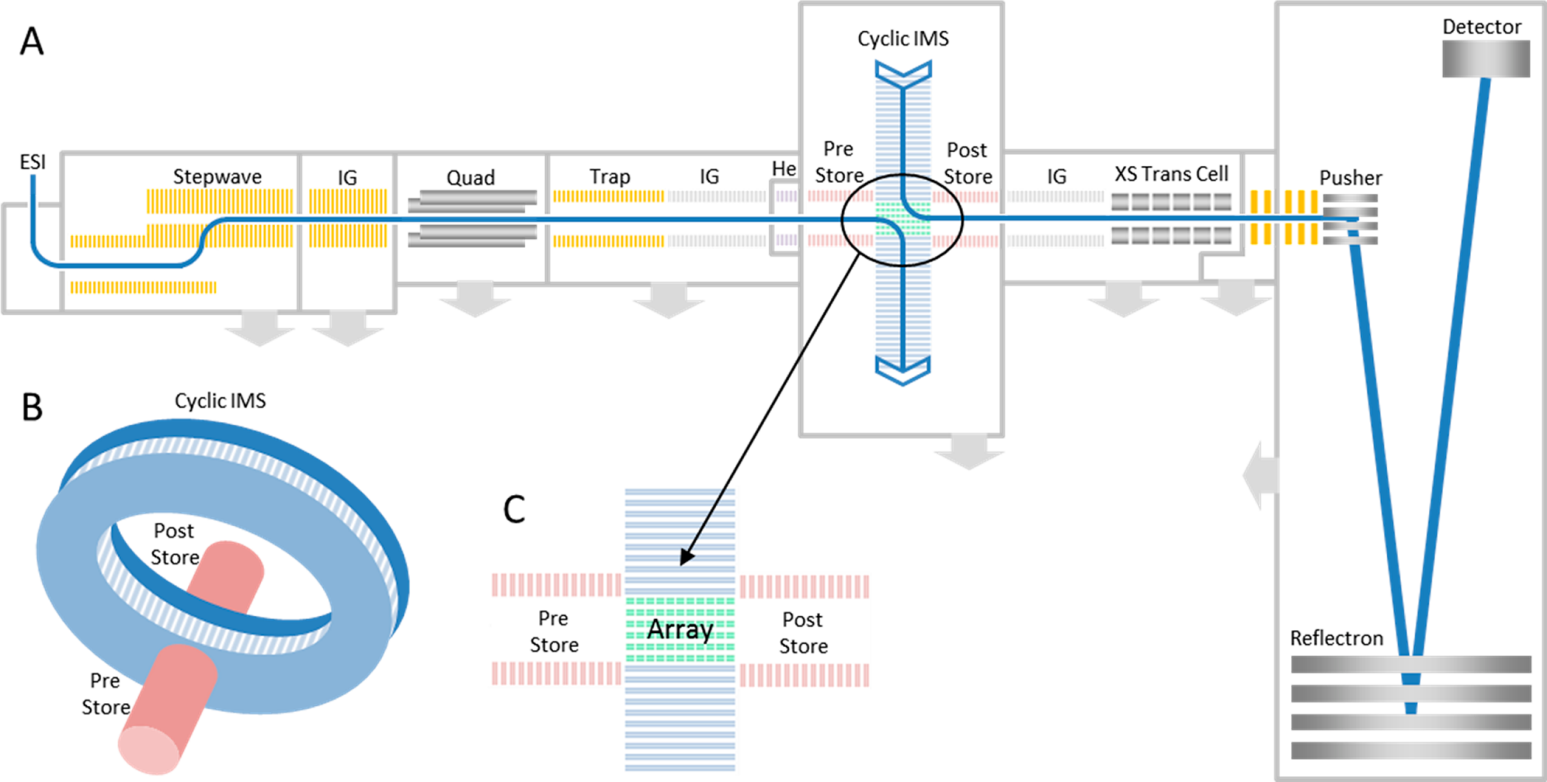
Schematic of cyclic IM QToF showing (A) the ion transmission pathway, (B) the set up of the cIM, and (C) the orientation of the array with respect to the pre- and poststore. Reproduced from ref (27). Copyright 2019 American Chemical Society.

**Figure 2:**
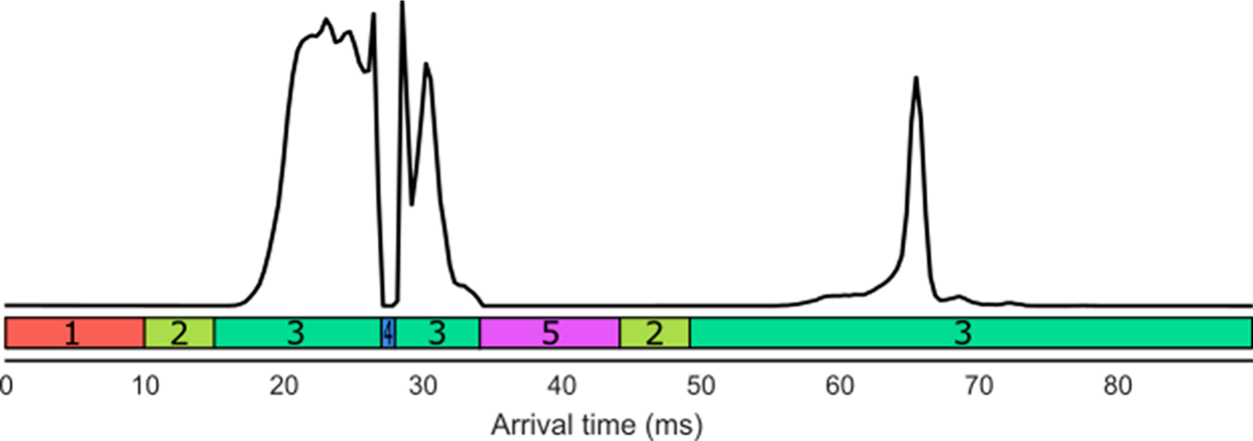
Representation of ATD of slice-CA, with corresponding functions for the cIM: 1, inject; 2, separate; 3, eject; 4, eject to prestore; 5, reinject from prestore. Reproduced in part from ref (27). Copyright 2019 American Chemical Society.

Samples were subjected to mobility selection and activation slice-CA as described in a previous publication where we touched on the activation of selected conformers in a limited fashion.^27^ Briefly, ions were injected into the cIM, underwent IM separation for a single pass, and were ejected from the cIM toward the detector (Figures 1 and 2). Ions of interest were ejected into the prestore rather than toward the detector and were reinjected into the array at varying activation energies once the other ions had been ejected. This subpopulation of ions was subjected to IM separation and analysis. Ions were also activated prior to IM selection, allowing us to study how transitions occur at different voltages (CA-IM-CA-IM rather than IM-CA-IM). For slice-CA parameters and injection sequences, see Tables S2–S4 and S8.

### Background Ion Signal

Background data collection was performed by keeping the initial slice-CA injection sequence parameters used for data collection and setting the “eject to prearray store” function to 0.01 ms so that a negligible quantity of ions are stored. To keep the injection sequence times the same, the “eject” function was increased to compensate for the shortened storing function (Figure S1). Each voltage increment of each slice and background was collected for 250 scans exactly.

### Ion Aging Experiments

Before each sequence of events occurring in the cIM, ions are accumulated in the trap ion guide (Figure 1) to ensure high duty cycle. To verify that conformational changes were not occurring during accumulation in the trap, ions were held there for varying times before a single pass separation, as described previously.^27^

### Data Analysis

Data were analyzed using Masslynx v4.1 (Waters Corp., UK), Driftscope v2.9 (Waters Corp, UK) and in-house software which allows multidimensional alignment and selection of the cyclic data (work in progress in our lab). CA fingerprint plots were created using benthesikyme.^29,30^ Population tracking was performed using an algorithm written in Python 2.7 (SI methods). For tracking using Gaussian functions, the second derivative was used initially to identify peak tops, and then populations were manually selected for good fit. Each peak center was fixed, and the height and width of each function was adjusted so that the sum of all functions has a low RMSD with data, as described previously (Figures S2–4 and SI methods).^27^ We attempted to use the minimum number of peaks possible while still maintaining low RMSD. Certain Gaussians were designated with a Greek letter if they appeared to correlate to a high intensity or major population. The peak top values of the designated Gaussian peaks are described in Table 1.

**Table 1:** Arrival Time Peak Top Values of the Named Populations to 1 dp

conformer	peak top (ms)	conformer	peak top (ms)
α	29.5	ζ	40.4
β	31.0	η	44.8
γ	32.5	θ	47.3
δ	33.5	ι	56.1
ε	35.4		

## Results

### CytC Unfolding

**Figure 3:**
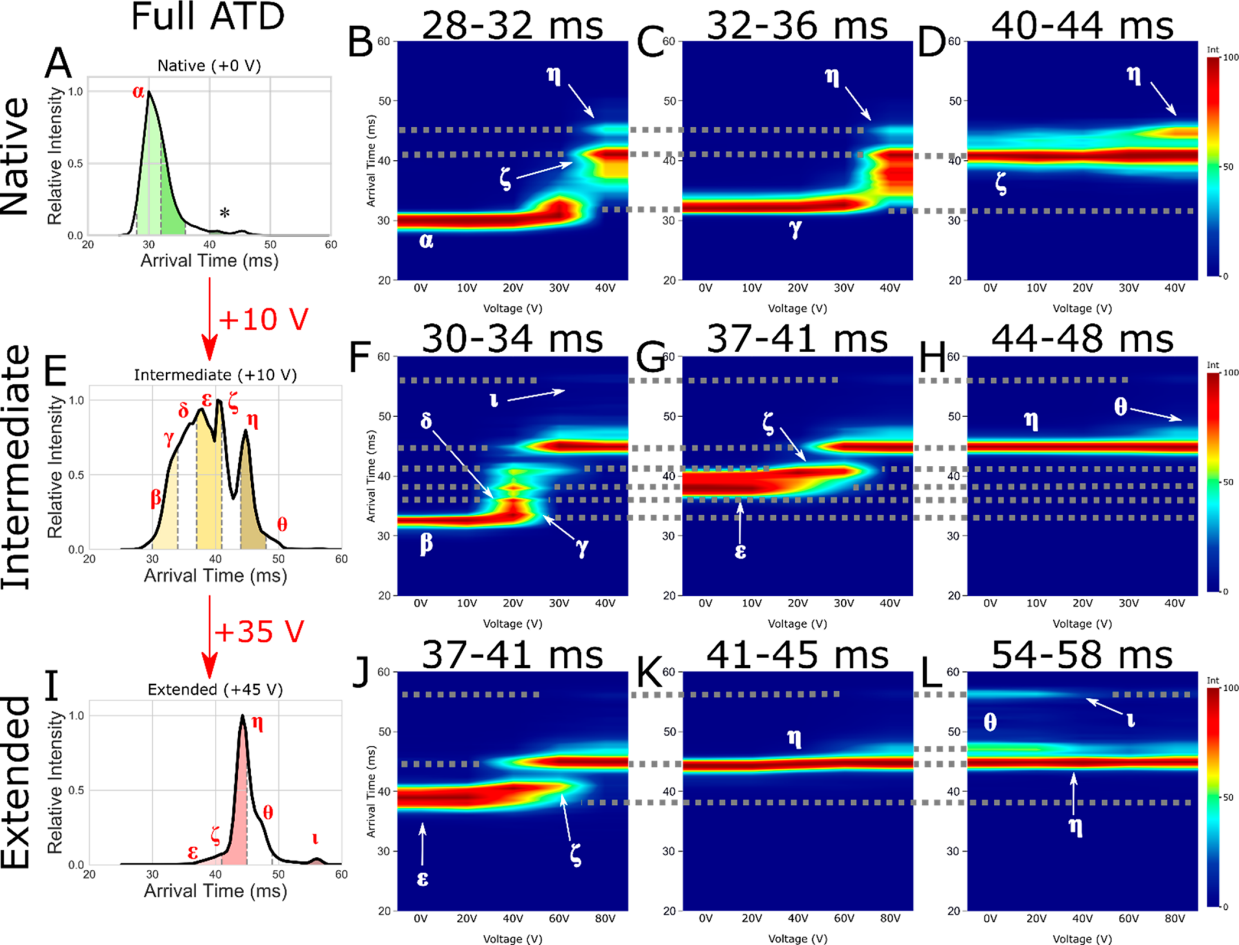
Arrival time distribution for (A) native (+0 V), (E) intermediate (+10 V), and (I) extended (an additional +35 V making +45 V total activation) CytC (+7) with subpopulation slices taken for CA denoted by dashed gray lines, filled with green (native), yellow (intermediate), and red (extended). Also shown are CA fingerprints for subpopulations of interest corresponding to native (B, F, J), intermediate (C, G, K), and extended (D, H, L) states, with interpopulation conformations joined by dotted gray lines. Identified conformations of interest are labeled as α, β, γ, δ, ε, ζ, η, θ, and ι. The * denotes the low intensity species mobility selected in plot (D).

The +7 charge state of CytC was quadrupole isolated and injected with varying levels of activation energy into the helium cell/cIM. Varying injection activation produced three conformational profiles: a native profile (+0 V), activated intermediate states (+10 V), and highly activated extended states (+45 V). After one pass around the cIM, arrival time distribution (ATD) slices of 4 ms (Figure 3A,E,I, green, yellow, and red slices, respectively, for native, intermediate, and extended) were ejected to prestore (Figure 1C) and then reinjected to induce further activation. Selected and activated ions were then subjected to one pass around the cIM.

**Figure 4:**
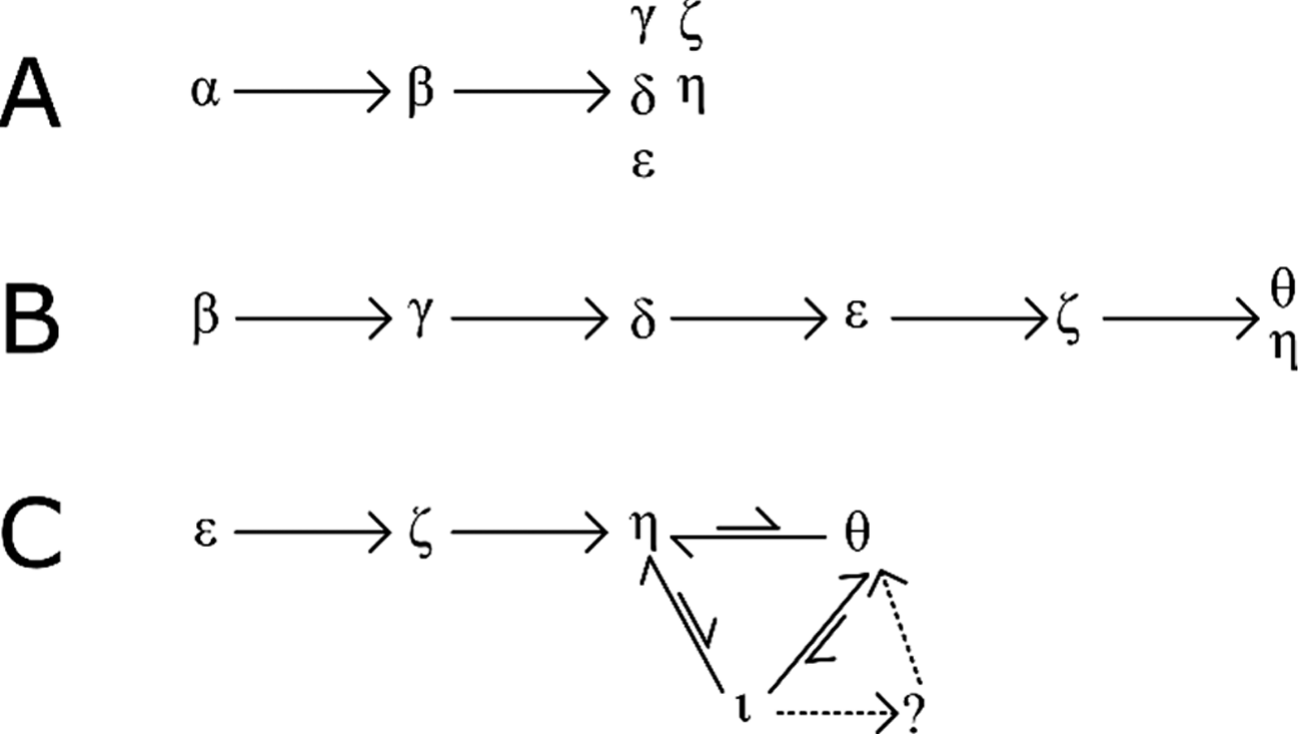
Transitional pathways of CytC gas phase unfolding.

The native ATD (Figure 3A, population α (29.5 ms)) presents as a single feature with an extended tail. This population of ions has been previously subjected to multiple passes around the cIM; however, we were not able to resolve them into more distinct features.^27^ Slice-CA overcomes this limitation and reveals a structural transition from α to a compact intermediate β (31.0 ms), which further transitions into multiple distinguishable intermediate conformational states (γ – η (32.5–44.8 ms), Figures 4A and S5). The activated intermediate population presents as a variety of features, some of which could be seen previously (α – η), and a new, low intensity population θ (47.3 ms) (Figures 3H and S6). Activation of these populations leads to direct conformational transitions from α – η and then to θ and ι (56.1 ms) (Figure 4B). The intensity of population η is low in comparison to the others and so is more easily visible in other plots (Figure S6).

**Figure 5:**
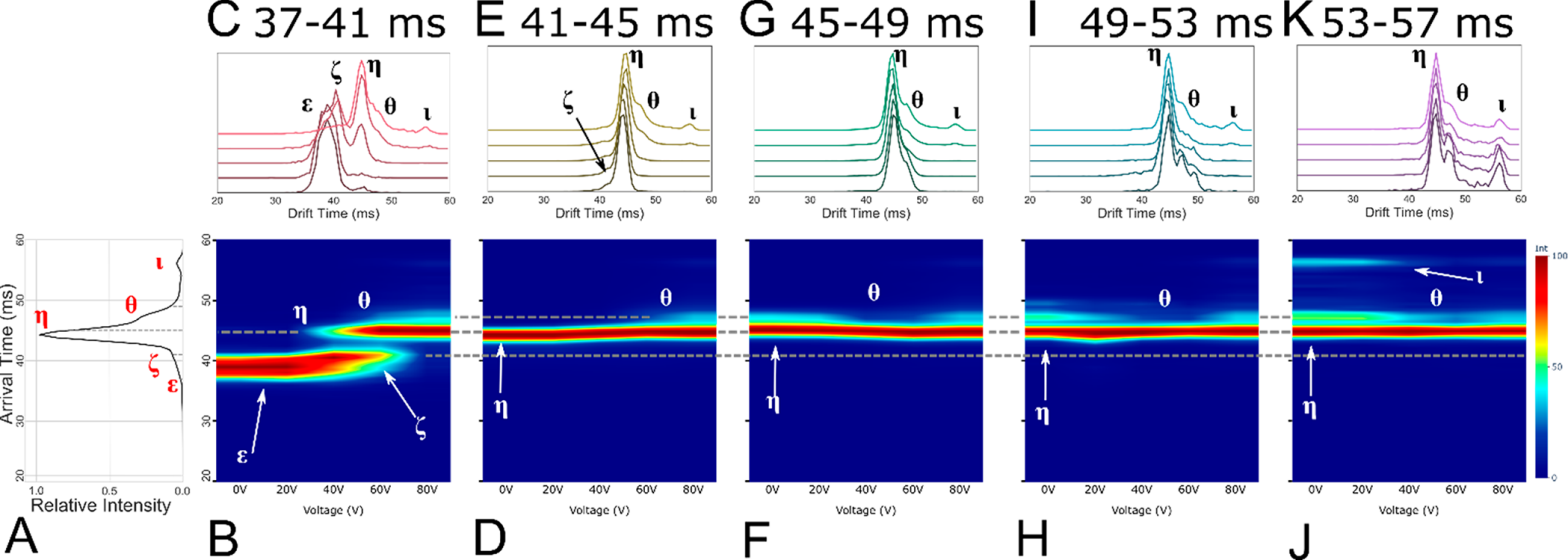
Full slice CA data for the +7 cytc extended state: (A) full ATD preslice, (B, D, F, H, J) CA fingerprint of each successive slice, (C, E, G I, K) stacked ATD plot of each slice with selected populations labeled ε, ζ, η, θ, and ι.

Activation of the extended states ε to η (Figures 3I–K and 5B–E) results in ATD profiles which resemble those obtained after activation of states of ε and ζ at lower energies (Figure 3G,H). These ATDs are qualitatively similar to those observed with single stage-IM instruments. Using the slice-CA workflow, we can now isolate and study transitions of population ι in detail. Following the isolation of ι (Figures 3L, 5J,K, and S7), we observe a mixture of η, θ, and ι, suggesting that interconversion from ι is occurring spontaneously or that there is minimal activation during the trapping and separation processes, which allows ι to revert back to η and θ. This is further evidenced by peak asymmetry, resulting in a “bridge” between the peaks of η, θ, and ι (Figures 5, S7, and S8). Under activation, the intensity of η drops with a corresponding rise in intensity of η and θ. We hypothesize that η and θ are highly stable kinetically trapped states, as evidenced by the very high energy required to induce a conversion from η to θ (Figure 4C). Interestingly, θ produced from ι appears as a sharper peak than is initially present as θ (Figures 4C and 5I,K). This may indicate other interconversion processes occurring in the conformational ensemble observed initially (causing peak asymmetry, broadening and “bridges”, Figures S7 and S8) or the presence of an “unresolved” intermediate (Figures 3I and 4C). This will be the focus of a future study.

### hIAPP Interconversion

hIAPP is a highly amyloidogenic 37-residue polypeptide neuropancreatic hormone produced by β-cells.^31−33^ In its monomeric state, hIAPP is a soluble, dynamically disordered polypeptide which samples a partial helical structure, but it forms islet amyloid in the pathogenesis of type 2 diabetes mellitus.^31,34^ Islet amyloid formation leads to β-cell dysfunction and death^35,36^ and contributes to the failure of islet cell transplantation.^31^ The mechanism of hIAAP amyloid formation is not understood; in particular, the early events in aggregation are opaque, and very little is known about the properties of low-order oligomers despite their importance in toxicity.

nESI mass spectra of hIAPP show a mixture of predominantly monomers and lower order oligomers with overlapping charge-state distributions (Figure S9). Species present at 1952 *m*/*z* for hIAPP were quadrupole selected and mobility separated, showing themselves to be two distinct conformers (Figure 5A). They were determined by isotopic peak spacing to be a mixture of +4 dimer (2*n*^+4^) and +2 monomer (1*n*^+2^, Figure 5B,C). The added dimension of mobility separation allowed for the selection and retention of the +4 dimer ion population in the instrument prestore for further investigation.

The +4 dimer species was divided into an early and late slice of 2 ms each and ejected into the prestore before reinjection into the cIM device at increasing voltages. At voltages between 30 and 60 V, both slices produced superimposable ATDs after activation, suggesting that the equivalent conformational ensemble was produced (Figure 5D). This conformational interconversion may be indicative of a low energetic barrier between the two states and is not observable using conventional single-stage IM experiments, where it appears as single-stage dissociation.^37^ Above 60 V, dimers begin to dissociate into monomers (Figure 5E,F).

This observation was not due to an increase in signal-to-noise (S/N) ratio during activation. Repeat experiments showed that this effect is robust between different instruments and samples. Measurement of “background” signal confirms this; the early, late, and background data were collected for exactly 250 scans and background ion counts could be seen; however, they were not present in the mobility space in which the hIAPP ions were present and were 3 to 4 orders of magnitude lower in intensity (Figure S10).

## Discussion

The experiments enabled by the Q-cIM-ToF instrument described here reveal insights into the conformational behavior of proteins in the gas phase and overcome the major limitation highlighted in our previous work, which is that increased resolution for ATDs produced from protein ions is limited due to the width and complexity of the conformers present.^27^ Application of the methodology to the native state of CytC allowed detection of low abundance conformational states and interconversion between them. Furthermore, the technique allowed mapping of the gas phase unfolding pathway of CytC allowing discrimination between irreversible and reversible unfolding transitions. Application of IM^n^ to selected extended states revealed interconversion involving three conformational states of differing stability, suggestive of low energy barriers between these states. We posit that the CytC + 7 extended conformers η and θ are kinetically trapped and thermodynamically stable as it requires high energies to induce a transition from between η and θ and that they can be formed from the more extended conformer ι. However, the transitions between ι to η/θ might occur if the ions have undergone thermal relaxation over the time-limits of IM selection. We think this unlikely considering that the activation of ι induces compaction. The possibility of the thermal relaxation of highly extended protein structures could be studied by aging the ions in the prestore instead of activating them. We anticipate this to be a topic of further study, but stabilization of transient conformers upon cooling has been seen a number of times in the gas phase.^38−40^

Currently, CA data are useful for comparison between distinct states, such as holo- and apoprotein, and measuring the comparative difference in stability imparted by ligand binding.^8^ While slice-CA experiments allow observation of conformational changes of selected subpopulations, further work will be required to extract energetic information from those data.^41,42^ A complementary approach for further development could involve conversion of collision energy to effective temperature via calibration using, e.g., variable temperature IM data or using “thermometer” ions.^41^ This could be correlated with molecular dynamics simulations at different temperatures to create models of existing ensembles in the gas phase.^43^

Studying the conformational dynamics of IAPP has been very difficult due to its dynamic nature and transient helical structure. Studies using circular dichroism and NMR are limited and are not able to capture the dynamics of the low-order oligomers, which are cytotoxic and undergo multiple conformational transitions before forming the amyloid fibril.^44−48^ Multiple single-stage IM studies have shown measurable differences between IAPP constructs^49−51^ However, previous single-stage IM studies on a series of single and double proline substitution constructs, which have different amyloidogenicities and cytotoxicity, could not define conformational differences within the resolution afforded by CCS, measurements. This highlights the need for higher resolution methods.^51^

**Figure 6:**
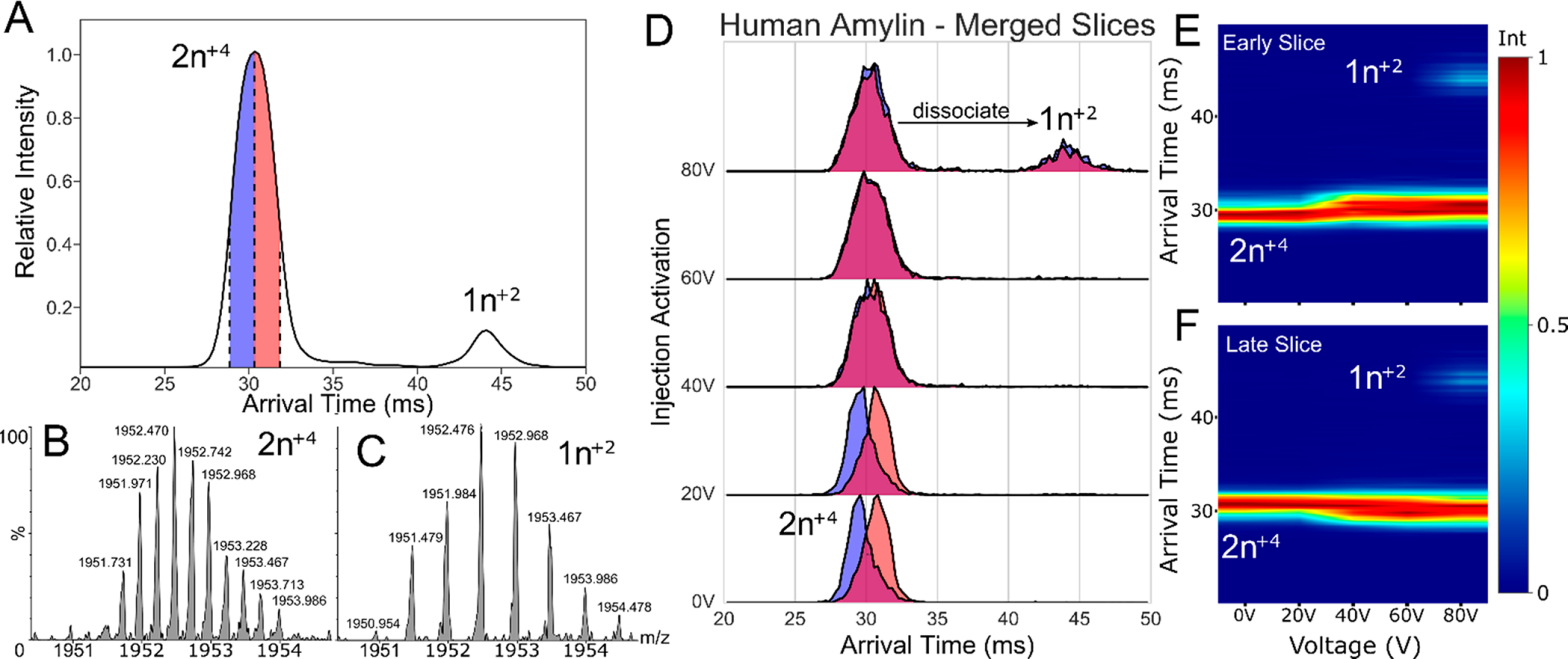
Slice-CA of hIAPP: (A) ATD of 1952 *m*/*z* species showing a mix of +2 monomer (1*n*^+2^) and +4 dimer (2*n*^+4^) where *n* is the oligomeric state; (B) isotopic peak envelope for dimer; (C) isotopic peak envelope for monomer; (D) stacked IM plots of early slice (blue) and late slice (red); (E) CA fingerprint of early slice; (F) CA fingerprint of late slice.

The interconversion observed here shows the potential of the slice-CA approach and may open a new avenue of study for dissecting the relationship between sequence, structure, and dynamics and the toxicity and amyloidogencity of hIAPP (Figure 6).^52^ In fact, an increasing number of studies point to the fact that proteins involved in misfolding and aggregation adopt different conformations with differences in the ability of these conformers to propagate, for example, the case with prions (i.e., different strains). Another example is provided by recent work with α-synuclein which has shown that different conformers exhibited distinct seeding and propagation properties, distinct cell killing abilities, and targeted different cell types.^53^ A major reason why such early conformers have not been widely studied, despite their importance, is because available biophysical techniques probe the ensemble average. The slice-CA method demonstrated here overcomes this key limitation by allowing the study of specific conformational subsets.

The results reported here demonstrate that the slice-CA methodology is able to distinguish between different types of protein gas-phase behavior such as sequential unfolding and interconversion of conformers, allows identification of reversible and irreversible unfolding transitions, and will therefore be of great importance in studies of protein dynamics and misfolding. We note that while the terminology “CIU” is commonly used in the field, our data show that unfolding does not always occur after activation. A similar observation has been made recently with the small peptide substance P.^54^ While substance P is much smaller than CytC or hIAPP, it highlights that the interconversion behavior we observed could be widespread. Also observed were the presence of conformers which were not selected for (Figure 3L), suggesting a degree of conformational flexibility in the gas phase. This points to the fact that protein ions may be not static under separation, and the conformers observed are likely dynamic. The approach demonstrated here overcomes these limitations. IM^2^ was used in this work; however, the geometry of the Q-cIM-ToF instrument allows multiple mobility selections to be performed which could reveal even finer details regarding the behavior of proteins during activation conditions in the gas phase. We anticipate the IM^n^ coupled with activation to become a popular tool as more tandem IM instruments become commercially available.

## Supporting Information Available

Detailed instrument parameter tables for collection of CytC and hIAPP data, population tracking for CytC, full slice-CA data sets for CytC, peptide synthesis methods, and population tracking methods (PDF)

## Abbreviations

ATD: arrival time distribution
CA: collision activation
CCS: collision cross-section
CID: collision-induced dissociation
CIU: collision-induced unfolding
CytC: cytochrome C
ESI: electrospray ionization
hIAPP: human islet amyloid polypeptide
IM: ion mobility
IM-MS: ion mobility mass spectrometry
IM^n^: tandem IM selection
MALDI: matrix-assisted laser desorption/ionization
MS: mass spectrometry
nESI: nano electrospray ionization
QToF: quadrupole time-of-flight
