# Supplementary material for: Cyclic Ion Mobility–Collision Activation Experiments Elucidate Protein Behavior in the Gas Phase
Source: J Am Soc Mass Spectrom. 2021 May 18;32(6):1545–52. doi: 10.1021/jasms.1c00018 (PMC8172447; doi:10.1021/jasms.1c00018)
Supplement: Supplementary file 1 — js1c00018_si_001.pdf [file js1c00018_si_001.pdf]

# Cyclic Ion Mobility – Collision Activation Experiments Elucidate Protein Behaviour in the Gas-Phase: Supporting Information

Charles Eldrid[1], Aisha Ben-Younis[1], Jakub Ujma[2], Hannah Britt[1], Tristan Cragnolini[3], Symeon Kalfas[1], Dale Cooper-Shepherd[2], Nick Tomczyk[2], Kevin Giles[2], Mike Morris[2], Rehana Akter[4], Daniel Raleigh[1,4] and Konstantinos Thalassinos[1,3]*

[1]Institute of Structural and Molecular Biology, Division of Biosciences, University College London, London, WC1E 6BT, UK, [2]Waters Corporation, Wilmslow, SK9 4AX, UK, [3]Institute of Structural and Molecular Biology, Birkbeck College, University of London, London, WC1E 7HX, UK, [4]Stony Brook University, 100 Nicolls Rd, Stony Brook, NY 11794, USA

*Corresponding author: k.thalassinos@ucl.ac.uk

| Parameter | Value |
|---|---|
| Capillary | 1.5 kV |
| Sampling Cone | 40 V |
| Twave height | 35 V |
| Twave velocity | 375 ($ms^{-1}$) |
| Trap CE | 5 V |
| Post Trap Bias | 25-60 V |

**Table S1**: Detailed data collection parameters for CytC

| Cyclic Function | Time (ms) | PreArrayGradient (V) | PreArrayBias (V) | Array Offset (V) | Array Twave Height(V) |
|---|---|---|---|---|---|
| Inject | 10 | 85 | 70 | 45 | 5 (forward) |
| Separate | 2 | 85 | 70 | 70 | 35 (sideways) |
| Eject | 14 - 29 | 85 | 70 | 45 | 25 (forward) |
| Eject to pre-store | 4 | 45 | 70 | 55 | 25 (backward) |
| Eject | 29 - 15 | 45 | 40 | 45 | 25 (forward) |
| Reinject from pre-store | 10 | 85 - 165 | 70 - 150 | 45 | 2 (forward) |
| Separate | 5 | 85 | 70 | 70 | 35 (sideways) |
| Eject and acquire | - | 85 | 70 | 45 | 25 (forward) |

**Table S2**: Cyclic function sequence for CytC Native (Figure 1A). For detailed description of cyclic functions please refer to Giles *et al.* *Anal. Chem.* 2019, 91, 13, 8564-8573.

| Function | Time (ms) | PreArrayGradient (V) | PreArrayBias (V) | Array Offset (V) | Array TWave Height (V) |
|---|---|---|---|---|---|
| Inject | 10 | 85 | 70 | 50 | 5 (forward) |
| Separate | 2 | 85 | 70 | 70 | 35 (sideways) |
| Eject | 13 - 25 | 85 | 70 | 45 | 25 (forward) |
| Eject to pre-store | 4 | 45 | 40 | 55 | 25 (backward) |
| Eject | 31 - 19 | 45 | 40 | 45 | 25 (forward) |
| Reinject from pre- store | 10 | 85 - 125 | 70 - 110 | 45 | 2 (forward) |
| Separate | 5 | 85 | 70 | 70 | 35 (sideways) |
| Eject and acquire | - | 85 | 70 | 45 | 25 (forward) |

**Table S3**: Detailed cyclic sequence for CytC Intermediate (Figure 1E).

| Cyclic Function | Time (ms) | PreArrayGradient (V) | PreArrayBias (V) | Array Offset (V) | Array Twave Height (V) |
|---|---|---|---|---|---|
| Inject | 10 | 85 | 70 | 45 | 5 (forward) |
| Separate | 2 | 85 | 70 | 70 | 35 (sideways) |
| Eject | 22 - 38 | 85 | 70 | 45 | 25 (forward) |
| Eject to pre-store | 4 | 45 | 70 | 55 | 25 (backward) |
| Eject | 6 - 22 | 45 | 40 | 45 | 25 (forward) |
| Reinject from pre-store | 10 | 85 - 165 | 70 - 150 | 45 | 2 (forward) |
| Separate | 5 | 85 | 70 | 70 | 35 (sideways) |
| Eject and acquire | - | 85 | 70 | 45 | 25 (forward) |

**Table S4**: Detailed cyclic sequence for CytC Extended (Figure 1I)

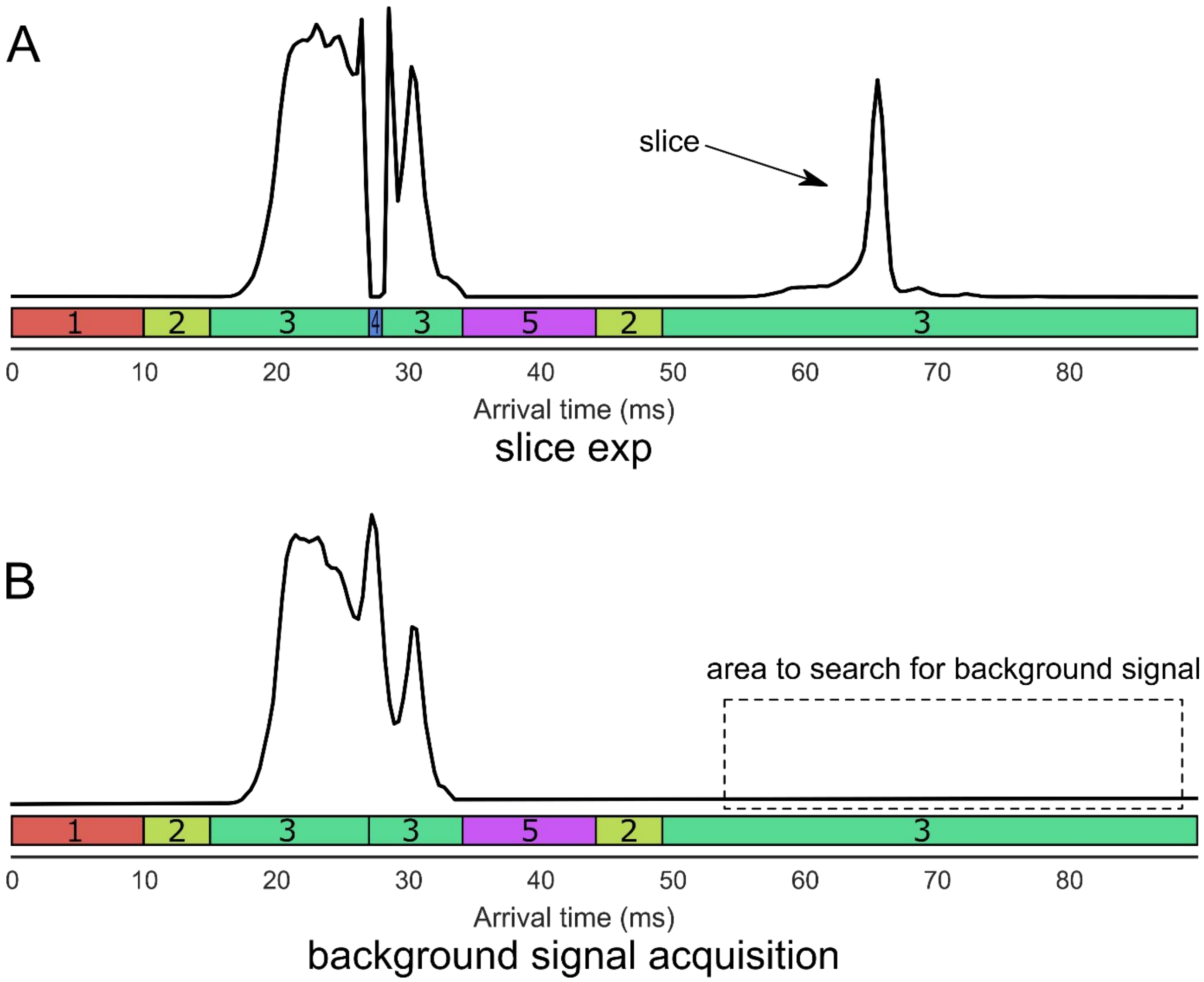


**Figure S1**: Representation of ATDs from background ion signal experiment. A) representative ATD from slice-CA experiment and injection sequence underneath B) representative ATD from collecting background injection signal. No ions are being injected to pre-store (function 4) but the reinject from pre-store function (function 5) allows identification of background ions.

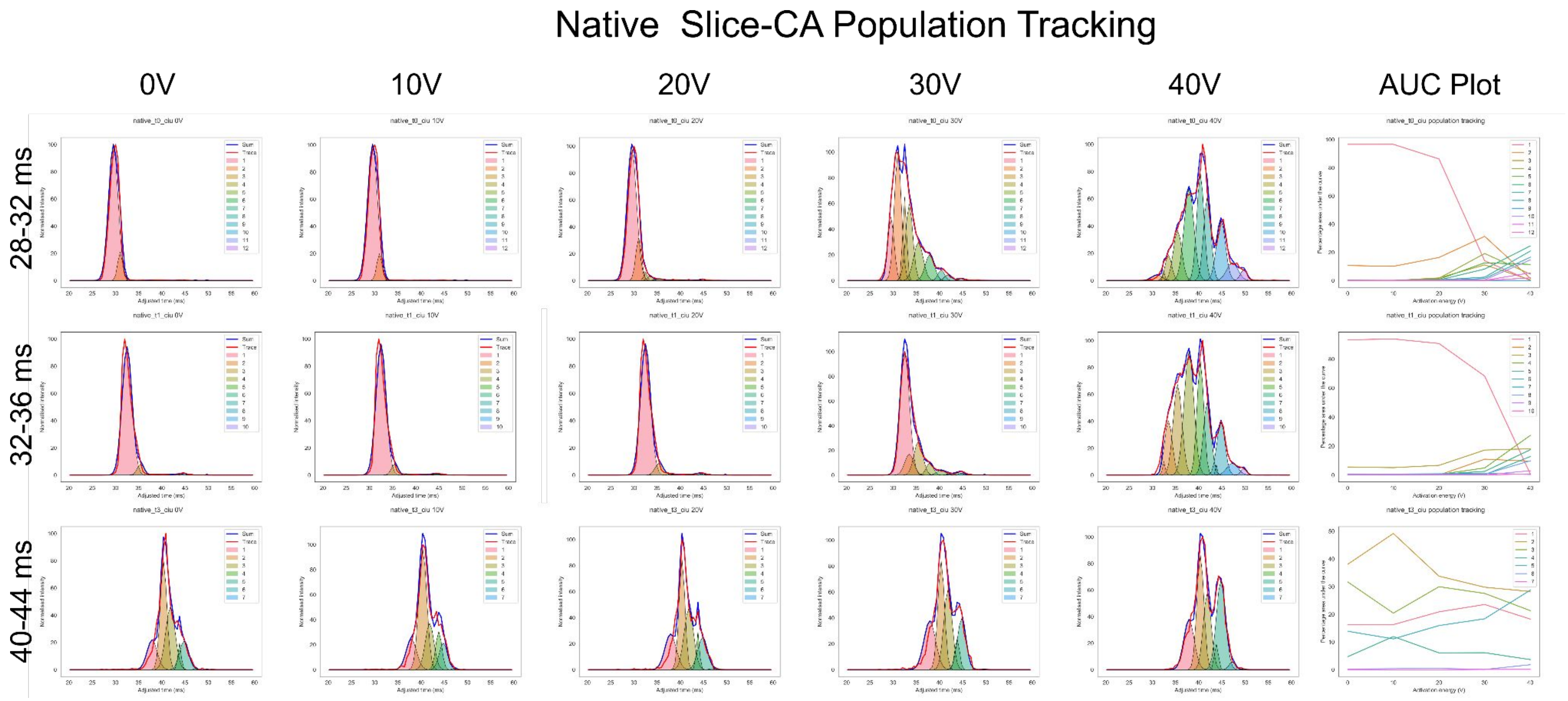


**Figure S2***:* Population tracking for cytc native slice-CA, for the 3 slices

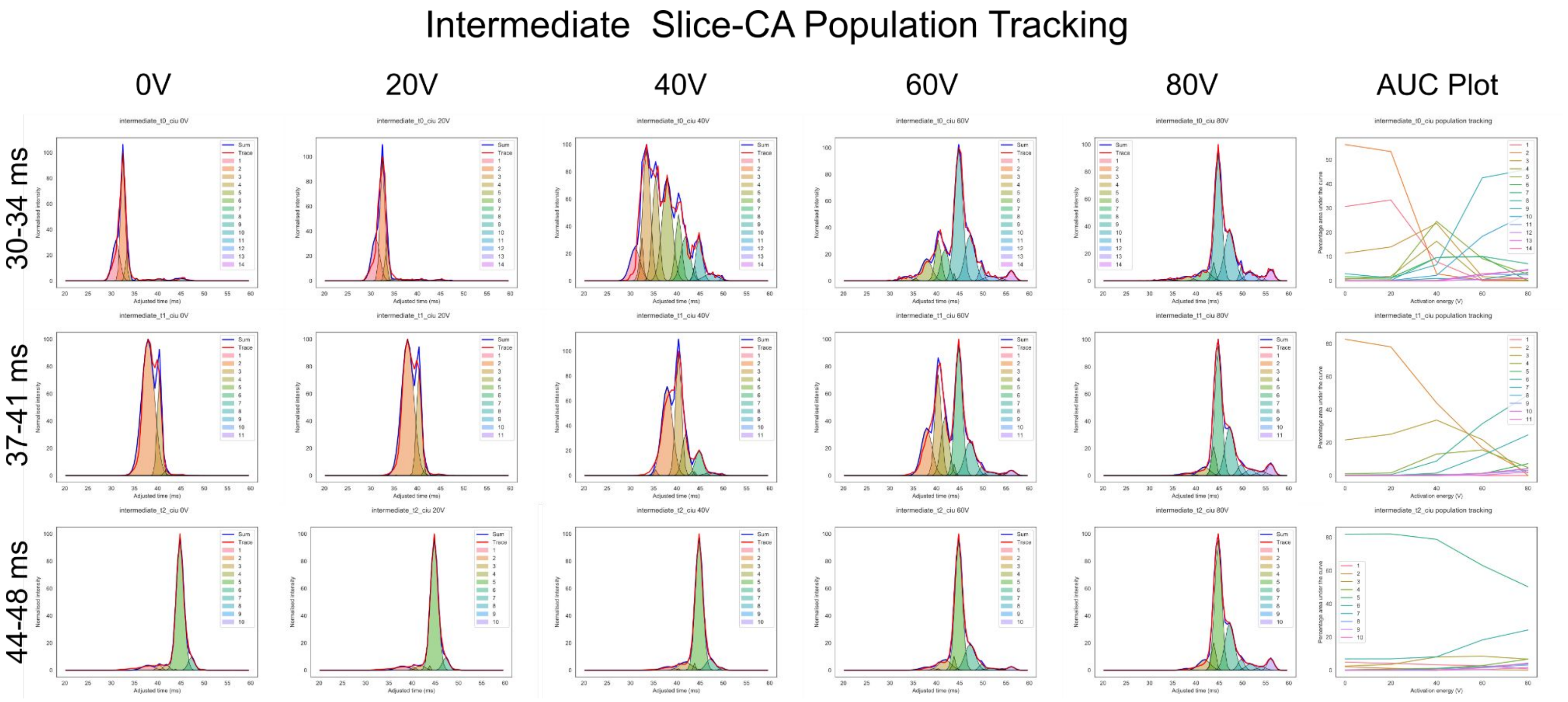


**Figure S3**: Population tracking for cytc intermediate slice-CA, for the 3 slices

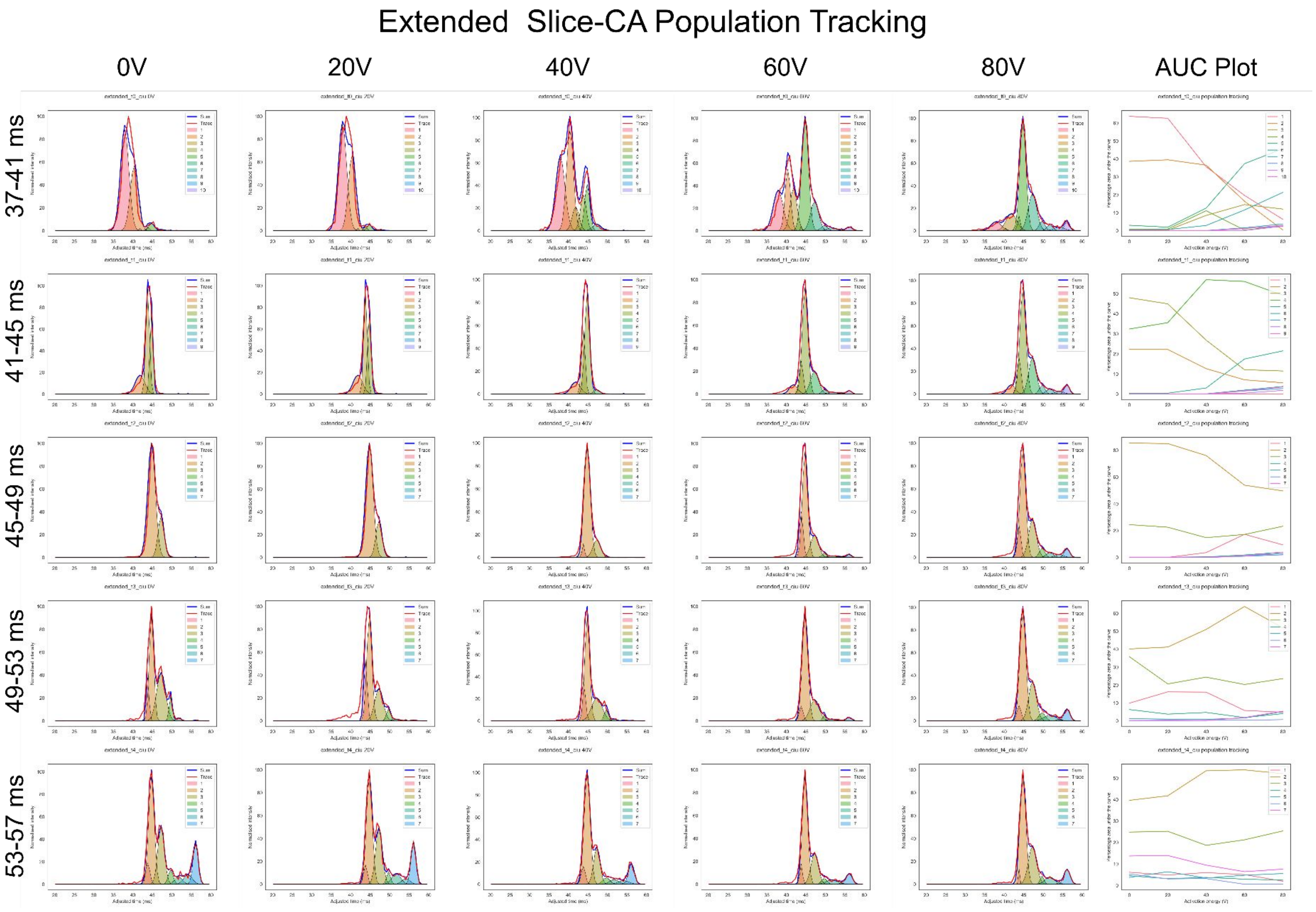


**Figure S4:** Population tracking for cytc extended slice-CA

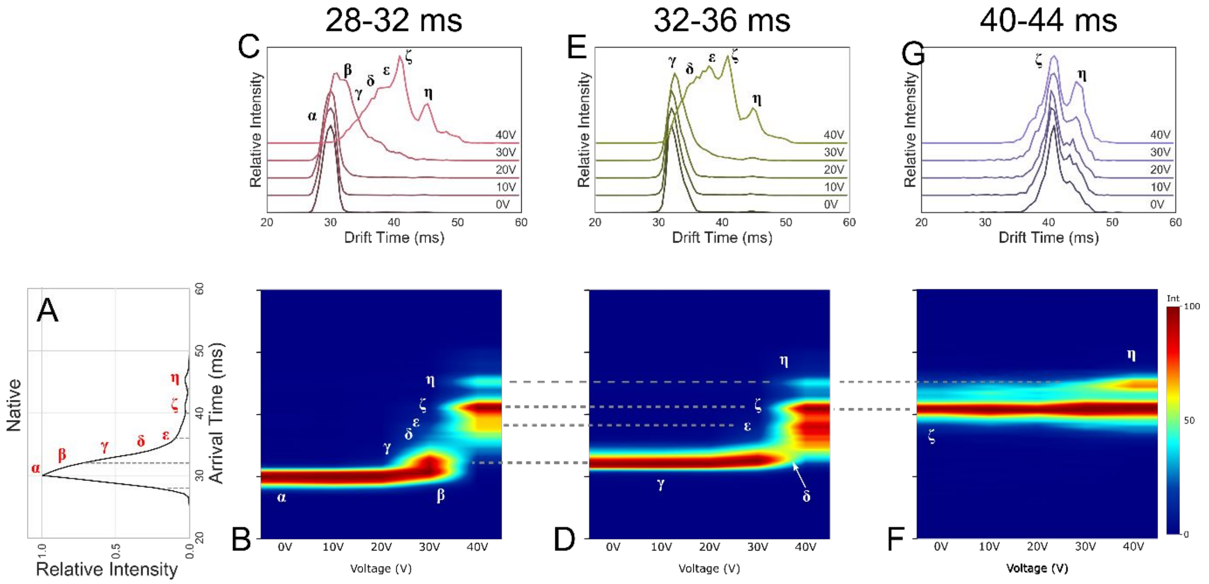


**Figure S5:** full slice CA data for the +7 cytc native state, A) full ATD pre-slice, B, D, F) CA fingerprint of each successive slice, C, E, G) stacked ATD plot of each slice with selected populations labelled α, β, γ, δ, ε, ζ and η.

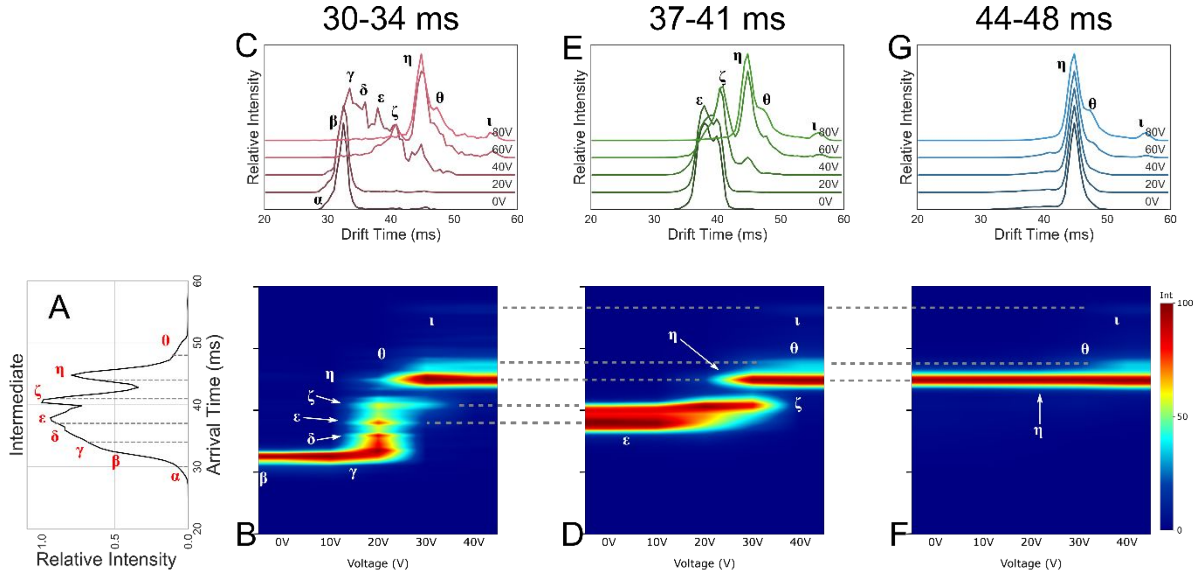


**Figure S6:** full slice CA data for the +7 cytc intermediate state, A) full ATD pre-slice, B, D, F) CA fingerprint of each successive slice, C, E, G) stacked ATD plot of each slice with selected populations labelled α, β, γ, δ, ε, ζ, η, θ and ι.

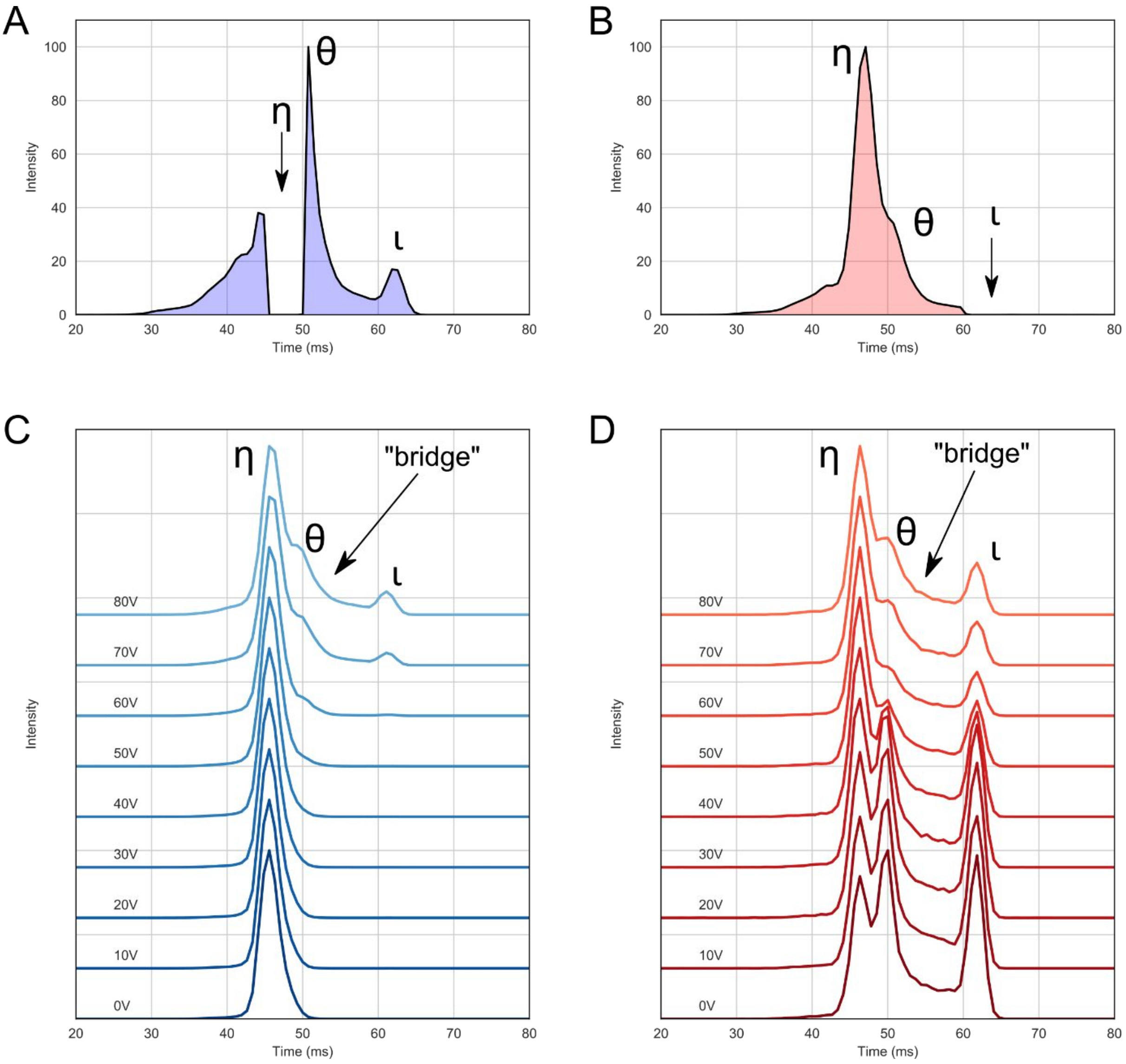


**Figure S7:** Detailed slice-CA of populations ε and η from extended +7 CytC. A) ATD with slice of population η removed B) ATD with slice of population ι removed C) Silce-CA of population η showing interconversion D) Slice-CA of population ι

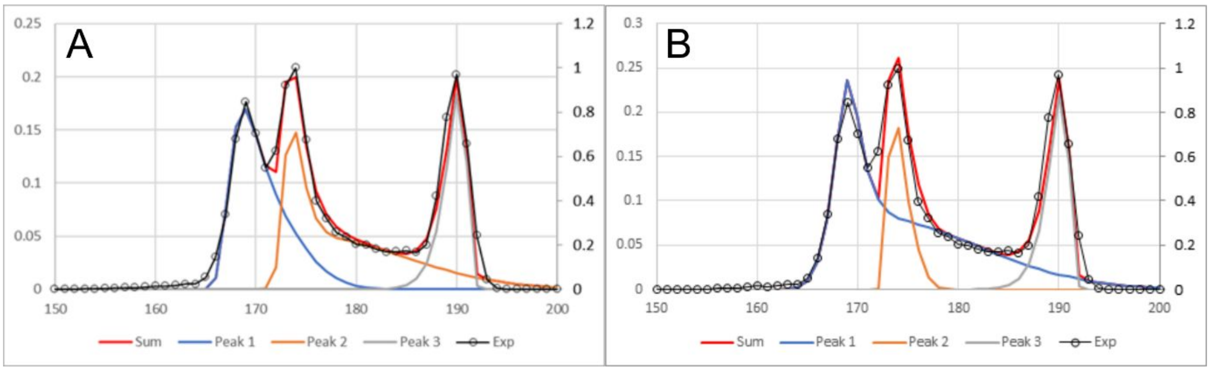


**Figure S8:** Theoretical depiction of assymetrical peak shape, rather than gaussian fitting presented in Figure S5, ie. "bridges" to explain the difference in peak shape of θ when produced from η or ι two different ATDs with a bridge are demonstrated here A) assymetrical peak of peak 1 (blue) and peak 2 (orange) and B) larger assymetrical peak 2

| Parameter | Value |
|---|---|
| Capillary | 1.3 kV |
| Sampling Cone | 20 V |
| Twave height | 30 V |
| Twave velocity | 375 ms-1 |
| Trap CE | 15 V |
| Post Trap Bias | 35 V |

**Table S7**: Detailed data collection parameters for hIAPP

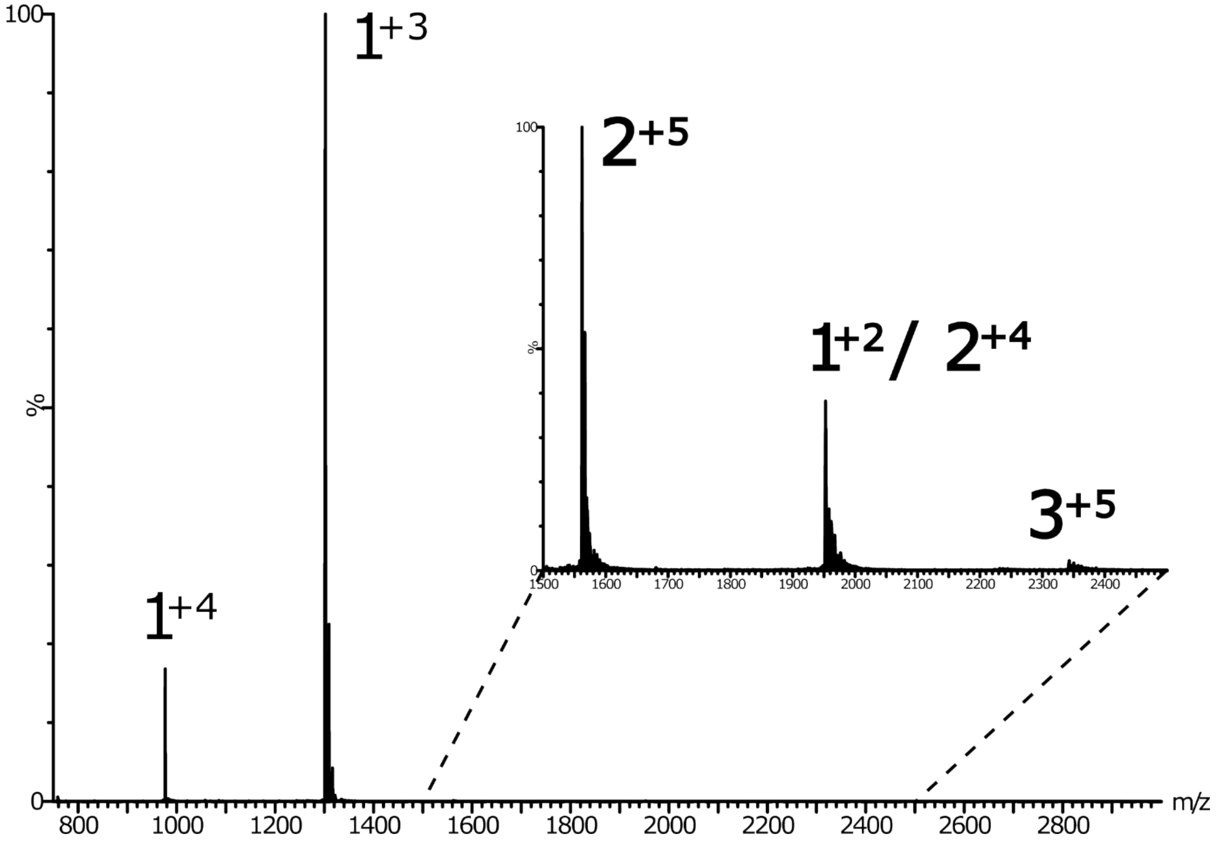


**Figure S9**: Full spectra of hIAPP, with the states mapped as oligomer (n) $n^{+z}$

| Function | Time (ms) | PreArrayGradient (V) | PreArrayBias (V) | Offset (V) 70 | Array Twave Height (V) |
|---|---|---|---|---|---|
| Inject (0) | 10 | 85 | 70 | 45 | 2 (forward) |
| Separate (1) | 5 | 85 | 70 | 67 | 35 (sideways) |
| Eject (14) | 22 - 20 | 85 | 70 | 45 | 25 (forwards) |
| Eject to pre-store (10) | 2 | 45 | 40 | 55 | 25 (backwards) |
| Eject (14) | 24 - 26 | 45 | 40 | 45 | 25 (forwards) |
| Reinject from pre-store | 10 | 80 - 160 | 65 - 145 | 45 | 2 (forwards) |
| Separate (1) | 5 | 85 | 70 | 45 | 35 (sideways) |
| Eject and acquire (6) | - | 85 | 70 | 45 | 25 (forwards) |

**Table S8**: Detailed cyclic sequence for hIAPP

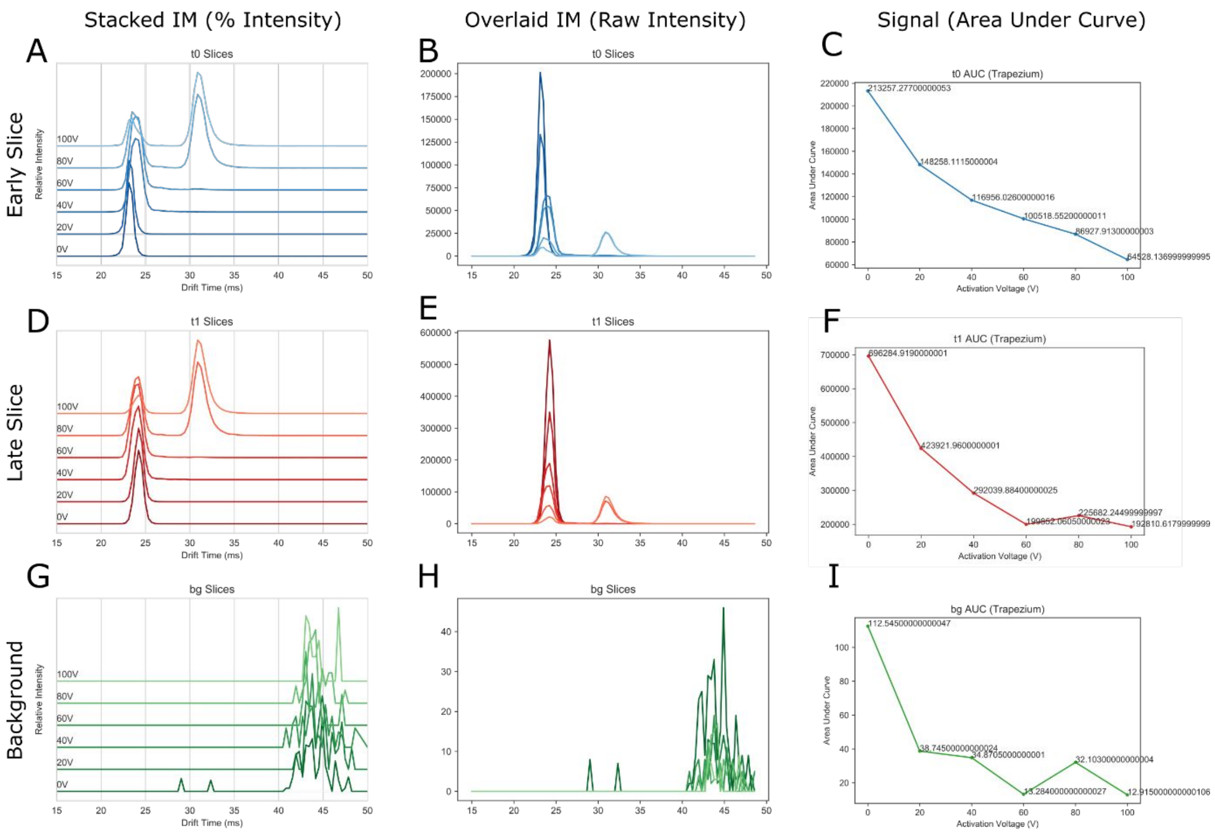


**Figure S10:** Analysis of signal counts so hIAPP slice-CA experiment shown in Fig 1. Here is shown the analysis for the early slice (A-C), the late slice (D-F) and background signal (G-I). Shown is the stacked IM plots of ATDs with relative intensity (A, D, G), the overlaid IM plots with the raw intensity (B, E, H) and the area under the ATD plots (with data being collected for each slice, for each voltage for 250 scans each) (C, F, I).

## Supplemental Methods

Peptide Synthesis and Purification

Human IAPP was using synthesized using 9-Fluorenylmethyloxycarbonyl (Fmoc) chemistry with a CEM Liberty Blue peptide synthesizer on a 0.10 mmol scale. Pseudoprolines derivatives were used as previously described to prevent aggregation during synthesis (1,2). The first residue attached to the resin, beta branched amino acids, arginine, and all pseudoproline dipeptide derivatives were double coupled. Fmoc-PAL-PEG-PS resin (0.18mmol/eq) was used to provide the naturally occurring C-terminal amide. The peptide was cleaved from the resin and side chain protecting groups removed using a trifluoroacetic acid (TFA) based cocktail (92.5% TFA, 2.5% triisopropylsilane, 3,6-Dioxa-1,8-Octanedithiol, and 2.5% $H_2O$) was used to cleave Crude, cleaved  peptide was dissolved in 20% acetic acid (4mg/mL) and lyophilized. The disulfide bond between residues Cys2 and Cys7 was formed by oxidation in 100% dimethyl sulfoxide (DMSO) at a concentration of 10mg/ml with gentle shaking at room temperature for three days. Human IAPP was purified via reverse-phase HPLC (Higgins Analytical C18 preparative column, 25mm x 250mm), utilizing gradient elution composed of buffer A (100% $H_2O$ and 0.045% HCl) and buffer B (80% Acetonitrile, 20% $H_2O$, and 0.045% HCl). Purified peptides were lyophilized. HCl was used as a counterion instead of TFA, as TFA can affect the rate of amyloid formation (3). A second HPLC purification was used to remove residual cleavage scavengers as well as residual TFA was used to dissolve the dry peptide was dissolved in 1,1,1,3,3,3-hexafluoroisopropanol and allowed to stand for several hours before the second purification.

1) Marek, P., Woys, A. M., Sutton, K., Zanni, M. T., and Raleigh, D. P. (2010) Efficient Microwave Assisted Synthesis of Human Islet Amyloid Polypeptide Designed to Facilitate The Specific Incorporation of Labeled Amino Acids, *Organic Letters 12*, 4848-4851.

2) Abedini, A., and Raleigh, D. P. (2005) Incorporation of Pseudoproline Derivatives Allows the Facile Synthesis of Human IAPP, a Highly Amyloidogenic and Aggregation-Prone Polypeptide, *Organic Letters 7*, 693-696.

3) Nilsson, M. R., and Raleigh, D. P. (1999) Analysis of amylin cleavage products provides new insights into the amyloidogenic region of human amylin, *Journal of Molecular Biology 294*, 1375-1385.

Gaussian Peak Fitting

Population tracking was performed using an algorithm using Python 2.7, available at: https://github.com/ThalassinosLab/CIVU. For population tracking using Gaussian functions initially the second derivative is used to identify peak tops and then populations were manually selected for good fit. Each peak centre is fixed and the height and width of each function is adjusted so that the sum of all functions has a low RMSD with data. The drift values (to 1 d.p.) used for each peak are as follows:

Native

| Time slice | Peak Centres (ms) |
|---|---|
| 28-32 ms | 29.5, 31.0, 32.5, 33.5, 35.4, 37.9, 40.4, 41.8, 43.8, 45.3, 47.3, 49.7 |
| 32-36 ms | 32.5, 33.5, 35.4, 37.9, 40.4, 41.8, 43.8, 45.3, 47.3, 49.7 |
| 40-44 ms | 33.5, 35.4, 37.9, 40.4, 41.8, 43.8, 45.3, 47.3, 49.7 |

Intermediate

| Time slice | Peak Centres (ms) |
|---|---|
| 30-34 ms | 31.0, 32.5, 33.5, 35.4, 37.9, 40.4, 41.8, 43.8, 44.8, 47.3, 49.7, 51.7, 54.1, 56.1 |
| 37-41 ms | 35.4, 37.9, 40.4, 41.8, 43.8, 44.8, 47.3, 49.7, 51.7, 54.1, 56.1 |
| 44-48 ms | 37.9, 40.4, 41.8, 43.8, 44.8, 47.3, 49.7, 51.7, 54.1, 56.1 |

| Time slice | Peak Centres (ms) |
|---|---|
| 37-41 ms | 37.9, 40.4, 41.8, 43.8, 47.3, 49.7, 51.7, 54.1, 56.1 |
| 41-45 ms | 40.9*, 41.8, 43.8, 47.3, 49.7, 51.7, 54.1, 56.1 |
| 45-49 ms | 43.8, 47.3, 49.7, 51.7, 54.1, 56.1 |
| 49-53 ms | 43.8, 47.3, 49.7, 51.7, 54.1, 56.1 |
| 53-57 ms | 43.8, 47.3, 49.7, 51.7, 54.1, 56.1 |

| Conformer | Arrival Time peak top (ms) | Conformer | Arrival Time peak top (ms) |
|---|---|---|---|
| α | 29.5 | ζ | 40.4 |
| β | 31.0 | η | 44.8 |

| | | | |
|---|---|---|---|
| γ | 32.5 | θ | 47.3 |
| δ | 33.5 | ι | 56.1 |
| ε | 35.4 | | |
